# Imaging of Proteinopathies in the Brains of Parkinsonian Disorders

**DOI:** 10.3390/cells14181418

**Published:** 2025-09-10

**Authors:** Makoto Higuchi

**Affiliations:** 1 Advanced Neuroimaging Center, Institute for Quantum Medical Science, National Institutes for Quantum Science and Technology, Chiba 263-8555, Japan; higuchi.makoto@qst.go.jp; 2 Neuroetiology and Diagnostic Science, Graduate School of Medicine, Osaka Metropolitan University, Osaka 545-8585, Japan

**Keywords:** frontotemporal lobar degeneration, multiple system atrophy, Parkinson’s disease, dementia with Lewy bodies, cross-β structure, cryo-electron microscopy

## Abstract

Neurodegenerative diseases such as Alzheimer’s disease (AD), frontotemporal lobar degeneration (FTLD), and α-synucleinopathies—including Parkinson’s disease (PD), dementia with Lewy bodies (DLB), and multiple system atrophy (MSA)—are characterized by the accumulation of misfolded protein aggregates. Advances in positron emission tomography (PET) imaging have enabled in vivo visualization of these pathologies, particularly tau and α-synuclein fibrils, facilitating early diagnosis and differential classification. Tau PET tracers such as ^18^F-florzolotau have demonstrated robust imaging of both AD-type and 4-repeat tauopathies, including atypical parkinsonian syndromes in FTLD such as progressive supranuclear palsy and corticobasal degeneration. Cryo-electron microscopy has elucidated the molecular interactions underlying tracer binding, highlighting hydrophobic grooves in cross-βstructures as binding components commonly present in multiple tau fibril types. For α-synucleinopathies, new tracers with a modified cross-β-binding scaffold, including ^18^F-SPAL-T-06 and ^18^F-C05-05, have shown promise in detecting MSA-related pathology and, more recently, midbrain pathology in PD and DLB. However, sensitive detection of pathologies in early PD/DLB stages remains a challenge. The integration of high-resolution PET technologies and structurally optimized ligands may enable earlier and more accurate detection of protein aggregates, supporting both clinical decision-making and the development of targeted disease-modifying therapies.

## 1. Introduction

Neurodegenerative diseases encompass a broad spectrum of clinical symptoms, including cognitive decline and motor dysfunction, and are pathologically characterized by the deposition of fibrous protein aggregates in the brain. The three major neurodegenerative dementias—Alzheimer’s disease (AD), frontotemporal lobar degeneration (FTLD), and dementia with Lewy bodies (DLB)—each exhibit distinct patterns of protein deposition. In AD, amyloid-β (Aβ) and tau proteins accumulate, whereas in FTLD, aggregates consist of either tau, TDP-43, or FUS [1]. The core pathology of DLB is the accumulation of α-synuclein, though Aβ and tau may also be co-deposited. FTLD includes several disease entities, among which progressive supranuclear palsy (PSP) and corticobasal degeneration (CBD) are associated with tau aggregation and frequently lead to parkinsonian syndromes [1]. Other α-synucleinopathies, such as Parkinson’s disease (PD) and multiple system atrophy (MSA), predominantly present with motor symptoms [2]. The characteristic motor features of PD include resting tremor, rigidity, bradykinesia, and postural instability—collectively referred to as parkinsonism. Given that DLB and MSA also commonly exhibit parkinsonism, a close association is presumed between α-synuclein pathology and motor dysfunction.

If protein aggregates such as tau and α-synuclein could be visualized in the living brain, patients with cognitive symptoms could be classified into AD, DLB, or FTLD, while those with predominant motor symptoms could be differentiated as having PSP, CBD, DLB, PD, or MSA. Thus, imaging of in vivo pathology may enable more precise disease diagnosis and differential classification. Furthermore, since protein aggregation is thought to be a major driver of neurodegeneration, efforts have intensified toward developing disease-modifying therapies targeting these aggregates [3]. Imaging technologies for visualizing pathological aggregates are therefore expected to play a pivotal role in determining treatment eligibility and evaluating therapeutic efficacy.

## 2. Imaging of Tau Pathology in Atypical Parkinsonian Disorders

Protein aggregates often form elongated fibrils composed of stacked β-sheet structures. By utilizing small molecules that bind specifically to these β-sheet assemblies, imaging of aggregates in the living brain becomes feasible. Positron emission tomography (PET) employs radiolabeled small molecules—commonly labeled with positron-emitting isotopes such as carbon-11 (^11^C) or fluorine-18 (^18^F)—enabling flexible chemical design. This has led to the successful development of PET probes for detecting Aβ and tau aggregates [1,4].

Tau imaging is particularly informative for diagnosing α-synucleinopathies such as DLB, PD, and MSA. Tau is a microtubule-associated structural protein that stabilizes the cytoskeleton of neuronal axons [5]. In AD, tau undergoes excessive phosphorylation, reducing its affinity for microtubules and promoting self-aggregation. This destabilization, along with the cytotoxicity of aggregated tau, contributes to neuronal degeneration [5]. While tau pathology in AD is predominantly neuronal, in PSP and CBD, tau also accumulates in glial cells [5]. In the central nervous system, alternative splicing of the tau gene yields six isoforms. In AD, all isoforms aggregate, whereas in PSP and CBD, only the 4-repeat (4R) isoforms form aggregates [5]. These differences influence the structural conformation of tau fibrils, and even between PSP and CBD—which share the same isoform composition—fibril structures differ. This structural heterogeneity underpins disease-specific localization at the cellular and regional level [6].

**Figure 1:**
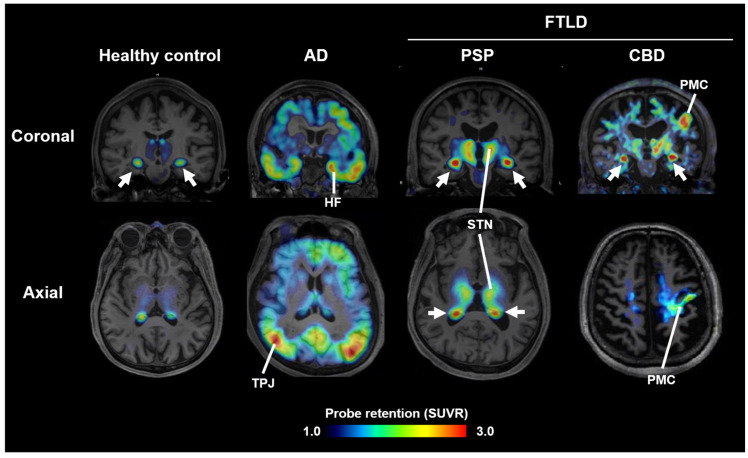
Tau PET images acquired with ^18^F-florzolotau in healthy controls and cases with AD, PSP, and CBD. Arrows indicate non-specific radioactivity accumulations in the choroid plexus. HF, hippocampal formation; STN, subthalamic nucleus and neighboring thalamic structures; PMC, primary motor cortex; TPJ, temporoparietal junction; SUVR, standardized uptake ratio at 90–110 min after tracer injection. Modified from Ref. [9].

Most PET probes in current clinical use bind preferentially to AD-type tau fibrils and show low affinity for 4R tau aggregates in PSP and CBD [7]. In contrast, the ^11^C-labeled compound PBB3, developed at National Institutes for Quantum Science and Technology (QST), binds broadly to structurally diverse tau fibrils [8]. However, due to rapid metabolism following intravenous injection, its brain uptake was suboptimal. To improve metabolic stability, ^18^F-florzolotau (also known as ^18^F-PM-PBB3 or ^18^F-APN1607) was developed. This probe allows high-contrast imaging of tau pathology in AD, PSP, and CBD (Figure 1) [9]. In PSP and CBD patients with pronounced parkinsonism, high uptake is observed in subcortical regions such as the midbrain, subthalamic nucleus, and basal ganglia (Figure 1), whereas PD patients show little to no uptake, and DLB patients display limbic and neocortical accumulation similar to AD. These differences in uptake patterns facilitate differential diagnosis among parkinsonian syndromes [9]. Notably, uptake in the subthalamic nucleus distinguishes PSP patients from healthy controls with ~95% sensitivity and specificity, and correlates with disease severity on the PSP rating scale [9,10].

Because the subthalamic nucleus is a small structure, quantification of a PET signal requires expert definition of the region of interest. Alternatively, AI-based pattern recognition using automated brain parcellation can provide diagnostic markers. When trained on tau PET images from AD and PSP patients, AI-generated scores for “AD-likeness” and “PSP-likeness” (termed AD-tau score and PSP-tau score) achieved >95% accuracy in distinguishing AD, PSP, and healthy controls [11]. Clinical trials of ^18^F-florzolotau for AD are currently in Phase 2 in the U.S., Japan, and Taiwan, led by APRINOIA Therapeutics under a license from QST. A Phase 3 trial for PSP is planned and aims for clinical implementation within two years.

The ultrastructural basis of interactions between PET probes and AD-type or non-AD-type tau fibrils remains incompletely understood. Recent advances in cryo-electron microscopy (cryo-EM) have revealed the three-dimensional structures of the fibril cores specific to individual tauopathies [6]. Utilizing this technology, florzolotau molecules bound to the fibril cores of AD-derived tau have also been visualized [12]. In the AD brain, tau aggregates primarily consist of paired helical filaments (PHFs) and straight filaments, both of which are composed of dimerized protofilaments. These protofilaments form a hollow structure with groove-like pockets, into which florzolotau binds by spanning across stacked β-sheets. These grooves are defined by peptide bond surfaces, allowing flat, elongated molecules that fit into narrow slits to bind effectively. Some grooves are flanked by gate-like hydrophilic side chains that interact with adjacent residues, opening the slit and thereby enhancing hydrophobic interactions with florzolotau [5,12]. In addition, florzolotau molecules may stack within the grooves in parallel orientations, further stabilizing their binding [12].

More recently, cryo-EM studies have elucidated the binding modes of flortaucipir analogs, such as GTP-1 and MK-6240, to AD-derived PHFs. In these filaments, the side chains of four amino acid residues form a well-defined pocket into which GTP-1 and MK-6240 insert [13,14]. In contrast, the fibrils of non-AD tauopathies, including those composed of 4-repeat or 3-repeat tau, do not exhibit such pocket-forming side chains, making binding of these ligands less favorable. Conversely, the hydrophobic grooves mentioned above are commonly present across various types of tau fibrils, which likely explains why florzolotau can bind to a broader range of tau aggregates.

## 3. Imaging of α-Synuclein Pathology

Similar to tau fibrils, α-synuclein aggregates also adopt β-sheet-rich fibrillar structures. While β-sheet conformations differ between Aβ, tau, and α-synuclein, tau PET probes exhibit some degree of cross-reactivity with α-synuclein aggregates. Among these, PBB3 and florzolotau have shown relatively high affinity for α-synuclein pathology, as suggested by fluorescence staining and autoradiography of brain sections from MSA patients [15]. In PET imaging using ^18^F-florzolotau, abnormal α-synuclein deposition in the striatum of MSA patients has been detected [16], although its performance in detecting lesions in other brain regions remains uncertain. The binding affinity of ^11^C-PBB3 and ^18^F-florzolotau for α-synuclein aggregates in brain homogenates from MSA and DLB patients is modest, with half-maximal inhibitory concentration (IC_50_) values around 50 nM [17], suggesting insufficient strength for practical PET imaging.

To overcome this issue, we modified the central linker of florzolotau to alter the torsion of its molecular scaffold, and developed a series of compounds (the C05 series) with higher affinity for α-synuclein aggregates, achieving IC_50_ values in the 2–3 nM range [17]. Among them, C05-05 demonstrated good brain permeability and favorable properties as a PET probe. In a mouse model with striatal inoculation of α-synuclein fibrils, in vivo two-photon laser microscopy showed that systemically administered C05-05 could detect pathology spreading to the cerebral cortex. PET studies further confirmed that ^18^F-labeled C05-05 (^18^F-C05-05) enabled visualization of propagating pathology in both mouse and marmoset models [17]. In marmosets, sequential PET imaging with ^18^F-C05-05 and a dopamine transporter probe captured the progression of pathology from the injection site to widespread brain regions, along with degeneration of the dopaminergic system [17].

In parallel with the development of C05-05, QST launched an industry-academia collaboration for α-synuclein PET probe development. In 2017, the Quantum Imaging Drug Discovery Alliance for Brain and Mind was established to develop PET imaging agents for brain diseases in cooperation with multiple pharmaceutical companies. Currently, eight domestic firms participate in this alliance. Within this framework, QST initiated the Synuclein PET Alliance (SPAL) and collaboratively developed candidate probes with Takeda Pharmaceutical Company Limited (Tokyo, Japan), Eisai Co., Ltd. (Tokyo, Japan), and Ono Pharmaceutical Co., Ltd. (Osaka, Japan). This pre-competitive initiative yielded a novel compound, ^18^F-SPAL-T-06, which demonstrated faster brain kinetics and lower background signal than ^18^F-C05-05 in animal models.

**Figure 2:**
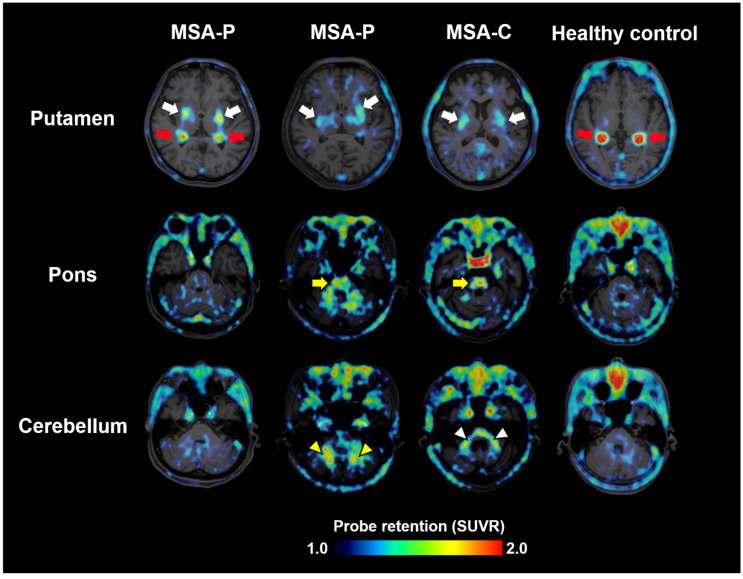
α-synuclein PET images acquired with ^18^F-SPAL-T-06 in healthy controls and cases with MSA-P and MSA-C. Red arrows indicate non-specific radioactivity accumulations in the choroid plexus. The probe captures pathologies in the putamen (white arrows), pons (yellow arrows), cerebellar peduncles (white arrowheads), and deep cerebellar white matter (yellow arrowheads). SUVR, standardized uptake ratio at 100–120 min after tracer injection. Modified from Ref. [18].

Clinical evaluation of ^18^F-SPAL-T-06 began in August 2021 in collaboration with the three aforementioned companies. PET scans were performed in three MSA patients and one healthy control. MSA includes two major subtypes: MSA-P, with predominant parkinsonism, and MSA-C, with cerebellar ataxia. The study included two MSA-P and one MSA-C patient. In all patients, increased probe accumulation was observed in multiple regions where α-synuclein pathology is known to occur, such as the basal ganglia, midbrain, pons, cerebellar peduncles, and deep white matter of the cerebellum (Figure 2) [18]. Notably, increased uptake in all these regions was observed regardless of MSA subtype, suggesting that the anatomical distribution of α-synuclein pathology does not always correlate with clinical phenotypes.

Further validation was performed using autoradiography and fluorescence staining of brain sections from MSA-P patients and healthy controls. These assays demonstrated that ^18^F-SPAL-T-06 specifically bound to brain regions rich in α-synuclein pathology and selectively labeled the oligodendroglial cytoplasmic inclusions characteristic of MSA [18]. In binding assays using brain homogenates, ^18^F-SPAL-T-06 showed high affinity for α-synuclein aggregates in MSA (IC_50_ ≈ 2.5 nM) and did not bind significantly to off-target molecules such as monoamine oxidases [18].

A radiotracer developed by AC Immune, ^18^F-ACI-12589, has been reported to detect α-synuclein deposits in the pons and cerebellar peduncles of patients with MSA [19]. However, its ability to visualize pathology in the basal ganglia appears limited, likely due to nonspecific accumulation of radioactivity in this region [19]. Similar to C05-05 and SPAL-T-06, ACI-12589 possesses a relatively extended molecular backbone, which is presumed to facilitate binding to the grooves within the cross-β structures of α-synuclein fibrils. Notably, this class of compounds can also bind to groove-like features in tau fibrils, leading to incidental labeling of tau aggregates on PET scans in patients with tauopathies.

**Figure 3:**
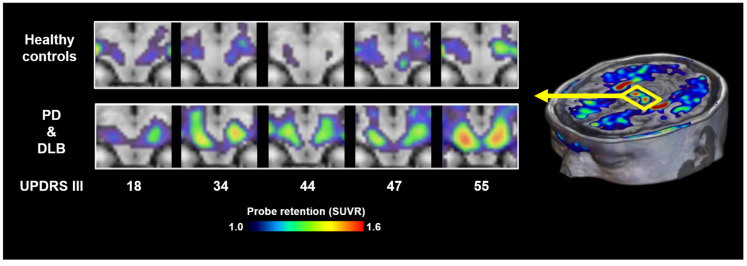
α-synuclein PET images acquired with ^18^F-C05-05 in the midbrains of healthy controls and cases with PD and DLB. MDS-UPDRS group III scores of the patients are attached to the bottom of the individual images. SUVR, standardized uptake ratio at 100–120 min after tracer injection. Modified from Ref. [17].

It is also noteworthy that neither ^18^F-SPAL-T-06 nor ^18^F-ACI-12589 has shown reliable visualization of Lewy bodies or Lewy neurites in patients with PD or DLB, possibly due to the relatively low density of these lesions compared to the abundant oligodendroglial cytoplasmic inclusions characteristic of MSA. More recently, ^18^F-C05-05 has been applied in clinical settings, and PET imaging has demonstrated its potential to detect α-synuclein pathology in the midbrain of PD and DLB patients (Figure 3) [17]. ^18^F-SPAL-T-06 and ^18^F-C05-05 displayed comparable affinity for α-synuclein aggregates in DLB brain homogenates but notably distinct pharmacokinetics in the brain [17,18]. ^18^F-SPAL-T-06 underwent much faster washout from the brain than ^18^F-C05-05, implying the possibility that more sustained concentrations of free ^18^F-05-05 in the brain increase the amount of radioligands accessible to the target molecules in the steady state. ^18^F-C05-05 retentions in the midbrain correlated with the severity of motor deficits assessed by MDS-UPDRS (Movement Disorders Society-sponsored Revision of the Unified Parkinson’s Disease Rating Scale) group III scores in PD and DLB cases [17]. However, this finding also indicates that there is no overt increase in radioligand binding at an early clinical stage of the disease.

Notwithstanding the advancement of clinically available PET probe development, the utility of α-synuclein imaging technology for monitoring disease progression or identifying individuals in the early stages of disease remains to be established. Development of next-generation α-synuclein imaging agents with enhanced sensitivity and specificity may benefit from further optimization of molecular features, such as the length and torsional flexibility of the compound backbone, to more precisely accommodate the conformational twist of binding grooves in α-synuclein fibrils.

As summarized in Table 1, several exploratory clinical PET studies have reported attempts to visualize α-synuclein pathologies using diverse tracers. For precise quantification of α-synuclein deposits in living individuals with early-stage PD or DLB, an optimal tracer would ideally exhibit a dissociation constant (K_D_) of <200 pM to elicit >20% increased uptake in affected brain regions. In addition to high affinity, the tracer should demonstrate rapid brain clearance, with a tissue half-life of approximately 20–30 min, to ensure sufficient contrast and dynamic imaging. Reducing background signal through optimized lipophilicity is also essential for enhancing image contrast. Crucially, off-target binding must be minimized. Prior studies have shown that avoiding interactions with monoamine oxidases A and B is necessary for improving tracer selectivity [18]. Furthermore, cross-reactivity with tau fibrils remains a major challenge, particularly for tracers targeting cross-β sheet structures common to various protein aggregates. To overcome this issue, alternative binding mechanisms are being explored. Recent studies have identified a new chemical class of compounds that selectively bind α-synuclein aggregates at non–cross-β sites [20], offering promise for the development of highly specific PET tracers for α-synuclein.

## 4. Comparative Utility of Proteinopathy PET and Conventional Neuroimaging Modalities in Parkinsonian Syndromes

While tau PET and α-synuclein PET offer molecular specificity by directly visualizing pathological protein aggregates, these methods are still emerging in clinical application. In contrast, established modalities such as dopamine transporter single-photon emission computed tomography (DAT-SPECT), ^123^I-metaiodobenzylguanidine (MIBG) scintigraphy, ^18^F-fluorodeoxyglucose positron emission tomography (FDG-PET), volumetric MRI, and diffusion tensor imaging (DTI-MRI) provide functional, autonomic, metabolic, and structural information. These methods often precede proteinopathy PET in clinical availability and offer complementary insights. Below, each modality in comparison to tau and α-synuclein PET is reviewed with respect to diagnosis, disease staging, and clinical decision-making.

(1) DAT SPECT

DAT-SPECT assesses presynaptic dopaminergic terminal integrity using ^123^I-FP-CIT (DaTSCAN). It is highly sensitive in detecting nigrostriatal degeneration and is used to differentiate PD or DLB from non-degenerative causes such as essential tremor, vascular parkinsonism, or drug-induced parkinsonism [24,25]. According to MDS diagnostic criteria, a normal DAT-SPECT scan constitutes an absolute exclusion for PD [26]. It also assists in differentiating DLB from AD in demented individuals.

However, because nigrostriatal degeneration is common to PD, DLB, PSP, and MSA-P, DAT-SPECT cannot differentiate these disorders [27]. Imaging studies have shown that striatal DAT binding is already reduced by 40–60% at symptom onset [28], and the decline may follow an exponential curve early in PD [29]. Hence, in terms of disease monitoring, the utility of DAT-SPECT remains limited. Notably, PET-based α-synuclein imaging has recently enabled the visualization of midbrain α-synuclein deposits, which may correlate with motor severity in PD and DLB [17]. However, it is yet to be clarified whether α-synuclein accumulation continues to increase linearly over the clinical course.

For PSP, there is currently no convincing evidence that DAT-SPECT is informative for tracking disease progression. By contrast, tau PET with ^18^F-florzolotau has demonstrated a robust correlation between subthalamic tau accumulation and clinical severity, as measured by PSP Rating Scale scores [9], highlighting the relevance of extra-striatal pathologies in PSP. Furthermore, recent findings suggest that tau PET can detect characteristic cortical and subcortical tau distributions associated with specific symptom domains in PSP [30]. These capabilities underscore a potential advantage of tau and α-synuclein PET over DAT-SPECT in capturing the broader neuroanatomical substrates of disease.

(2) MIBG scintigraphy

Myocardial scintigraphy using ^123^I-MIBG, a physiological analog of noradrenaline, assesses postganglionic sympathetic innervation. Degeneration of cardiac sympathetic nerve terminals, due to α-synuclein aggregation, leads to markedly reduced MIBG uptake in approximately 80–90% of PD cases [31]. Unlike current PET tracers, which are not yet capable of imaging α-synuclein pathology in the peripheral nervous system, MIBG scintigraphy uniquely reflects systemic autonomic involvement. This makes it particularly useful in differentiating PD and DLB, which exhibit sympathetic denervation, from atypical parkinsonian disorders such as MSA, PSP, and CBD, which typically retain normal or only mildly reduced MIBG uptake [32].

According to meta-analyses, MIBG scintigraphy can differentiate PD from MSA and PSP with 80–90% sensitivity and specificity [33]. Although newer PET tracers such as ^18^F-florzolotau for tauopathies [19] and ^18^F-SPAL-T-06 for MSA [18] may outperform MIBG in distinguishing specific diseases, MIBG remains a valuable modality that enables broad differentiation across multiple diagnostic categories with a single scan.

(3) FDG-PET

FDG-PET is a functional neuroimaging modality that measures regional cerebral glucose metabolism, primarily reflecting synaptic activity while also being influenced by glial activation. It is highly sensitive to early functional disturbances that may precede structural atrophy. In PD, FDG-PET has revealed disease-specific metabolic network patterns. Notably, a PD-related pattern characterized by hypometabolism in the posterior parietal and premotor cortex with relative preservation of the sensorimotor cortex has demonstrated >95% sensitivity and 70–80% specificity in distinguishing PD from MSA and healthy controls [34,35]. However, standardization across cohorts remains a challenge for widespread diagnostic implementation of such network-based assessments.

FDG-PET is also a well-established tool for differentiating DLB from AD. The “cingulate island sign”, which is preserved glucose metabolism in the posterior cingulate cortex amid occipital hypometabolism, is highly specific to DLB and aids in distinguishing it from AD [36]. Despite the growing use of tau and α-synuclein PET imaging, the precise relationships between regional protein deposition and FDG-PET-derived metabolic patterns remain unclear in parkinsonian disorders. Further studies integrating proteinopathy PET and FDG-PET may help clarify how pathologies affect functional brain networks over time.

(4) Volumetric MRI

Volumetric MRI enables quantitative analysis of brain structure using techniques such as voxel-based morphometry and automated segmentation to detect regional atrophy patterns. While conventional MRI is essential for excluding secondary causes (e.g., tumors, stroke), volumetric MRI adds value by capturing subtle, disease-specific atrophy patterns in parkinsonian disorders [37].

Although its diagnostic utility in idiopathic PD remains limited, volumetric MRI can help differentiate PD from atypical parkinsonian syndromes such as PSP and MSA. In particular, regional atrophy in the midbrain, cerebellum, or cortical regions improves the diagnostic yield, with reported positive predictive values approaching 90% [38,39]. However, volumetric MRI alone is insufficient to reliably distinguish between PD, CBD, PSP, and MSA with high accuracy. This highlights the need for protein-specific imaging such as tau and α-synuclein PET for more precise phenotypic discrimination. Recent comparative studies suggest that atrophy observed on MRI may reflect both local and network-mediated neurotoxicity driven by tau accumulation, as evidenced by corresponding tau PET findings [30].

(5) DTI

DTI is an advanced MRI technique that assesses microstructural integrity of white matter by measuring water diffusion along fiber tracts. Key metrics include fractional anisotropy (FA) and mean diffusivity (MD), which respectively reflect directional coherence and overall diffusion. In PD, DTI can detect early changes in the substantia nigra, typically reduced FA and increased MD, sometimes preceding volumetric atrophy [40]. However, these findings are often inconsistent across studies [41]. Despite its sensitivity, DTI has technical limitations, such as low resolution of crossing fibers and vulnerability to partial volume effects. Emerging approaches like diffusion kurtosis imaging and q-space diffeomorphic reconstruction aim to improve accuracy but require further validation in large cohorts [42].

In tauopathies and α-synucleinopathies, DTI abnormalities likely reflect axonal damage associated with pathological protein deposition and propagation. Supporting this, a recent study combining DTI and tau PET found that altered DTI metrics in PSP were observed in cortical tracts linking regions of tau accumulation and atrophy (Goto et al., unpublished data).

A critical challenge shared by all imaging modalities, including proteinopathy PET, is the low availability of a universally accepted standard of truth (SoT). Most studies use clinical diagnosis as the SoT, but this is suboptimal; neuropathological confirmation remains the gold standard. Indeed, the diagnostic accuracy of idiopathic PD in clinical practice has been reported to range from 75–80%, based on clinico-pathological studies [43]. Although conventional MRI can assist in excluding secondary causes, volumetric changes in the midbrain substantia nigra may support diagnosis of PD and DLB. However, the correlation of such changes with autopsy-confirmed pathology has not been systematically validated. Quantitative susceptibility mapping can detect nigral iron accumulation and showed moderate diagnostic accuracy (AUC = 0.74) for separating Lewy pathology-positive from negative cases using neuropathology as the SoT [44]. Autopsy-based studies indicate that distinct regional atrophies (e.g., in MSA-C and PSP) offer high specificity (~100%) but only moderate sensitivity (60–85%) for disease differentiation compared to controls [38].

Network-based classifications of FDG-PET have demonstrated rather high accuracy, correctly identifying 80% of PD and atypical parkinsonism cases when compared to postmortem diagnoses [45]. DAT-SPECT shows excellent sensitivity: retrospective studies of autopsy-confirmed PD cases reported 100% sensitivity [46,47]. However, these studies did not estimate specificity. Later, a study comparing DLB and AD found 86% diagnostic accuracy (sensitivity 80%, specificity 92%) relative to postmortem findings [48], although differentiation from atypical parkinsonism remains more challenging. Few studies have correlated proteinopathy PET findings with autopsy results in parkinsonian diseases. Single-case reports have demonstrated strong associations between in vivo ^18^F-florzolotau retention and regional tau pathology in frontotemporal lobar degeneration subtypes, including PSP [49] and Pick’s disease [50], while larger-scale validation studies are needed. Once neuropathological validation is established, tau and α-synuclein PET may serve not only as diagnostic tools but also as reference standards for evaluating other imaging modalities in the absence of postmortem data.

Neuroimaging-based indicators, in conjunction with fluid biomarkers, may enable a biologically grounded classification of PD. Recently, the SynNeurGe (synuclein + neurodegeneration + genetic) and SDG (synuclein + dopaminergic + genetic) frameworks have been proposed to categorize PD and related neuronal α-synucleinopathies such as DLB [51,52]. These classification schemes remain under refinement, particularly in enhancing sensitivity for early-stage pathology. To expand such frameworks across the broader spectrum of parkinsonian disorders, incorporation of tau biomarkers is essential. This would allow the detection of tau co-pathology in DLB and the delineation of primary tauopathies such as PSP and CBD. Moreover, the advent of PET tracers capable of visualizing glial α-synuclein aggregates in MSA holds promise for differentiating distinct subtypes within the parkinsonian spectrum. The integration of a multi-biomarker assessment strategy is expected to enhance the precision of patient recruitment and stratification of individuals with parkinsonism, while also supporting robust evaluation of therapeutic efficacy in clinical trials for primary tauopathies and α-synucleinopathies.

## 5. Conclusions

The development of PET probes capable of visualizing 4R tau and α-synuclein aggregates has opened new possibilities for the differential diagnosis of diseases presenting with parkinsonism, based on underlying molecular pathology. This represents a major breakthrough in molecular imaging technologies. These imaging modalities are also expected to facilitate patient selection and therapeutic monitoring for emerging antibody and nucleic acid-based disease-modifying therapies targeting tau and α-synuclein [53].

Since these treatments are likely to be more effective when initiated early, before irreversible neuronal loss occurs, it is essential to develop imaging methods that can sensitively detect initial aggregate deposition in small deep brain regions—such as tau accumulation in the subthalamic nucleus or α-synuclein aggregation in the substantia nigra. In collaboration with Atox Co., Ltd. (Tokyo, Japan), QST has developed a dedicated head-only PET scanner with high spatial resolution [54]. After regulatory approval, this system has been commercially available since 2022. With approximately twice the spatial resolution of conventional systems, the device enables clear visualization of small subcortical structures and is expected to facilitate early detection of protein aggregate pathology.

The integration of α-synuclein PET into the biological classification of PD and related disorders represents a critical next step, particularly when combined with dopaminergic and neurodegenerative biomarkers to build comprehensive SynNeurGe and SDG frameworks. Its unique role as the “Syn” or “S” component within these frameworks warrants further investigation, especially in comparison with emerging peripheral α-synuclein assays, such as those based on biological fluids [55,56] and tissue biopsies [57,58].

## Figures and Tables

**Table 1:** Clinically investigated PET tracers for imaging α-synuclein pathology. T_1_/_2_ indicates the half-life of brain tracer concentration following peak uptake. BKG @ 60 min denotes background radioactivity in the brain, expressed as a percentage of the peak uptake, measured at 60 min post-injection. * ^11^C-BF-227 was originally developed as an amyloid-β imaging agent. ** ^11^C-PM-PBB3 was originally developed for tau imaging. PK, pharmacokinetics; eIND, exploratory investigational new drug study; N/A, not assessed.

Name	Affinity	Brain PK		Off-Target Binding	Cross-Reactivity with Tau		Development Stage	References
		T_1/2_	BKG @ 60 min		Affinity	In Vivo Binding		
^18^F-C05-05	IC_50_ = 1.5 nM	~40 min	~35%	TMEM106B	IC_50_ = 12.9 nM	PSP	eIND for PD, DLB, MSA	[17]
^18^F-SPAL-T-06	K_D_ = 2.5 nM	~20 min	~25%	TMEM106B	N/A	N/A	eIND for MSA	[18]
^18^F-ACI-12589	K_D_ = 17–30 nM	~20 min	~30%		N/A	AD(, PSP)	eIND for MSA	[19]
^11^C-MODAG-005	K_D_ = 0.2 nM	~35 min	~45%		K_D_ = 7.1 nM		eIND for MSA	[21]
* ^11^C-BF-227	K_D_ = 46.0 nM	~40 min	~45%		Aβ K_D_ = 15.7 nM	AD Aβ	(MSA)	[22,23]
** ^11^C-PBB3	IC_50_ = 58.8 nM	~20 min	~35%	TMEM106B	IC_50_ = 8.6 nM	AD, PSP	(MSA)	[8,17]
Disired ligand	K_D_ < 0.2 nM	20–30 min	<25%		K_D_ > 50 nM			

## Data Availability

No new data were created or analyzed in this study.

## Abbreviations

Aβ	Amyloid-β
AD	Alzheimer’s disease
CBD	Corticobasal degeneration
Cryo-EM	Cryo-electron microscopy
DAT-SPECT	Dopamine transporter single-photon emission computed tomography
DLB	Dementia with Lewy bodies
DTI	Diffusion tensor imaging
FDG-PET	^18^F-fluorodeoxyglucose positron emission tomography
FTLD	Frontotemporal lobar degeneration
K_D_	Dissociation constant
MDS	Movement Disorders Society
MIBG	^123^I-metaiodobenzylguanidine
MSA	Multiple system atrophy
PD	Parkinson’s disease
PET	Positron emission tomography
PHF	Paired helical filament
PSP	Progressive supranuclear palsy
QST	National Institutes for Quantum Science and Technology
UPDRS	Unified Parkinson’s Disease Rating Scale
